# Sleep disorders in stroke rehabilitation: mechanisms, impacts, and multidisciplinary management — a narrative review

**DOI:** 10.3389/fneur.2026.1899982

**Published:** 2026-09-08

**Authors:** Guojin Hu, Xiuli Sun, Cheng Xie

**Affiliations:** Department of Geriatric Rehabilitation, Shanghai Second Rehabilitation Hospital, Shanghai, China

**Keywords:** cognitive behavioral therapy, CPAP, obstructive sleep apnea, rehabilitation, rTMS, sleep disorders, stroke

## Abstract

**Background:**

Sleep disorders and stroke share a bidirectional relationship. Sleep disturbances are independent stroke risk factors and frequent post-stroke complications. Obstructive sleep apnea (OSA), through intermittent hypoxia, systemic inflammation, endothelial dysfunction, and autonomic dysregulation, increases stroke risk by approximately 2- to 3-fold. Post-stroke sleep disorders affect up to 59.9% of patients, impeding neurological recovery.

**Objectives:**

To examine the epidemiology, pathophysiology, and clinical impacts of sleep disorders on stroke recovery, and to summarize evidence-based management strategies.

**Methods:**

A narrative review was guided by a writing outline developed from clinical observations and preliminary synthesis. A theme-driven literature search was conducted in PubMed, Embase, Cochrane, Web of Science, CNKI, and Wanfang (March 10–13, 2026) using targeted keywords (e.g., “sleep disorders,” “obstructive sleep apnea,” “rehabilitation,” “stroke”), limited to 2000–2026 publications in English or verified Chinese translations. Reference lists were also screened.

**Results:**

Post-stroke sleep disorder prevalence ranges from 25 to 84%; OSA affects 40–78% of stroke patients. Sleep disorders correlate with worse functional outcomes, prolonged hospitalization, cognitive decline, and elevated stroke recurrence. Mechanistically, intermittent hypoxia, oxidative stress, systemic inflammation, glymphatic dysfunction, autonomic dysregulation, and impaired neuroplasticity are implicated. Management: CPAP is first-line for OSA; CBT-I improves insomnia symptoms; rTMS has been shown to improve sleep quality in clinical studies; exercise, melatonin, and light therapy show emerging evidence. Pharmacotherapy for RLS/PLMD requires caution regarding fall risk and cognitive side effects.

**Conclusion:**

Sleep disorders are highly prevalent and negatively impact stroke recovery. Evidence-based strategies—CPAP, CBT-I, and rTMS—improve sleep and functional outcomes, though evidence heterogeneity exists. High-quality RCTs are needed to optimize individualized protocols.

## 1 Introduction

Stroke remains the second leading cause of death and the third leading cause of disability worldwide, imposing substantial burdens on individuals, families, and healthcare systems (1, 2). Despite advances in acute stroke management, approximately 70–80% of survivors experience varying degrees of motor dysfunction, cognitive impairment, and other neurological sequelae that significantly affect quality of life and social participation (3). Among these post-stroke complications, sleep disorders have garnered increasing attention due to their high prevalence and substantial impact on neurological recovery.

Stroke-related sleep disorders (SSDs) refer to a group of clinical syndromes that first appear after a stroke or that are pre-existing before a stroke but persist or worsen after the stroke, meeting the diagnostic criteria for sleep disorders (1). Based on the chronological order of the stroke and the sleep disorder, SSDs are divided into two types: post-stroke sleep disorders and stroke-associated sleep disorders (4).

The main types of sleep disorders include insomnia, sleep-disordered breathing (SDB), rapid eye movement sleep behavior disorder (RBD), excessive daytime sleepiness (EDS), restless legs syndrome (RLS)/periodic limb movements of sleep (PLMS), and circadian rhythmic sleep–wake disorders (CRSWDs) (5).

The relationship between sleep disorders and stroke is characterized by its bidirectionality. On one hand, sleep disturbances such as obstructive sleep apnea (OSA), insomnia, and restless legs syndrome (RLS) independently increase the risk of incident stroke through multiple pathophysiological pathways (6, 7). On the other hand, cerebrovascular events frequently disrupt sleep architecture and precipitate or exacerbate sleep disorders (8, 9). This intricate interplay creates a vicious cycle where sleep disturbances worsen stroke outcomes while stroke itself perpetuates sleep problems, ultimately hindering rehabilitation efficacy.

Among various sleep disorders, OSA demonstrates the strongest and most consistent association with stroke. Epidemiological evidence indicates that OSA affects approximately 34 and 17% of middle-aged men and women in the general population, respectively, while prevalence rates surge to 40–78% among stroke patients (10, 11). The Sleep Heart Health Study and Wisconsin Sleep Cohort Study have demonstrated that OSA is associated with a 2–3 fold increased risk of stroke after adjusting for conventional vascular risk factors (12, 13). Beyond OSA, other sleep disorders including insomnia, hypersomnia, parasomnias, and sleep-related movement disorders also demonstrate elevated prevalence in stroke populations and contribute to adverse neurological outcomes (14, 15).

The mechanisms linking sleep disorders to stroke encompass multiple physiological systems. Intermittent hypoxia resulting from recurrent airway obstruction in OSA has been shown to trigger oxidative stress, systemic inflammation, endothelial dysfunction, and sympathetic activation in experimental and clinical studies, pathways that may collectively promote atherosclerosis and thrombogenesis (16, 17). Furthermore, sleep fragmentation and disruption of slow-wave sleep impair glymphatic clearance of neurotoxic metabolites, including amyloid-β and tau proteins, thereby exacerbating neuronal injury following cerebral ischemia (18, 19). Conversely, stroke-related brain lesions can disrupt sleep–wake regulation centers, alter neurotransmitter systems, and precipitate sleep architecture abnormalities.

The clinical implications of post-stroke sleep disorders are multifaceted. Accumulating evidence suggests that untreated sleep disturbances are associated with worse functional outcomes, longer hospital stays, greater cognitive decline, higher depression rates, and increased risk of recurrent cerebrovascular events (20–22). A prospective cohort study demonstrated that sleep-disordered breathing accounted for 9–10% of ethnic disparities in functional and cognitive outcomes among stroke patients (20). Moreover, experimental studies in rodent models have revealed that sleep disruption following cerebral ischemia significantly aggravates brain damage and impairs endogenous repair mechanisms including neurogenesis, synaptogenesis, and angiogenesis (23).

Despite the recognized importance of sleep disorders in stroke care, these conditions remain frequently underdiagnosed and undertreated in clinical practice. Multiple barriers impede routine screening and management, including limited awareness among healthcare providers, lack of standardized assessment protocols, diagnostic challenges in post-stroke populations, and uncertainty regarding optimal treatment timing and strategies (24, 25). Addressing these knowledge gaps is essential for improving stroke care quality and rehabilitation outcomes.

This narrative review aims to synthesize current evidence regarding sleep disorders in stroke populations, covering epidemiological findings, pathophysiological mechanisms, impacts on recovery outcomes, and available therapeutic interventions. By summarizing the state of knowledge in this domain, we seek to inform clinical practice and identify priority areas for future research.

## 2 Search strategy and literature selection

This section describes the search strategy and literature selection process in sufficient detail to permit replication, addressing the limitations of vague methodological reporting in the original manuscript. This review is explicitly a narrative review, not a systematic review, and therefore does not employ a single predefined protocol registered *a priori*. The approach followed a PRISMA-inspired framework to ensure transparency and reproducibility.

### 2.1 Search databases and date

Electronic searches were conducted across six databases: PubMed/MEDLINE, Embase, Cochrane Library (Cochrane Reviews and Trials), Web of Science, CNKI (China National Knowledge Infrastructure), and Wanfang Data. The search was performed between March 10 and March 13, 2026. The time frame was restricted to publications from January 1, 2000, through March 10, 2026. No language restrictions were applied for English-language databases; Chinese-language searches in CNKI and Wanfang were limited to studies with English abstracts available or verified translation.

### 2.2 Search strategy and keywords

Rather than a single search string, theme-specific keyword combinations were developed for each section of the review, consistent with narrative review methodology. Core keyword themes and representative search strings are summarized below Table 1.

**Table 1:** Core keyword themes and representative search strings.

Theme	Keywords	Databases
Sleep disorders + stroke prevalence	*“sleep disorders” OR “sleep disturbance” AND “stroke” AND “prevalence”*	PubMed, Embase, Cochrane, WoS, CNKI, Wanfang
Obstructive sleep apnea	*“obstructive sleep apnea” OR “OSA” OR “sleep-disordered breathing” AND “stroke” OR “cerebrovascular”*	PubMed, Embase, Cochrane, WoS
Insomnia post-stroke	*“insomnia” OR “sleep quality” OR “PSQI” AND “stroke” OR “cerebrovascular accident”*	PubMed, Embase, Cochrane, WoS, CNKI
Restless legs syndrome	*“restless legs syndrome” OR “RLS” OR “periodic limb movement” AND “stroke”*	PubMed, Embase, WoS, CNKI, Wanfang
Pathophysiology mechanisms	*“intermittent hypoxia” OR “glymphatic” OR “neuroinflammation” OR “autonomic” AND “stroke” OR “sleep apnea”*	PubMed, Embase, Cochrane, WoS
CPAP therapy	*“CPAP” OR “continuous positive airway pressure” AND “stroke” OR “rehabilitation”*	PubMed, Embase, Cochrane, WoS
CBT-I and behavioral therapy	*“cognitive behavioral therapy” OR “CBT-I” AND “insomnia” AND “stroke” OR “brain injury”*	PubMed, Embase, Cochrane, WoS
Acupuncture and TCM	*“acupuncture” OR “electroacupuncture” OR “traditional Chinese medicine” AND “stroke” OR “sleep”*	PubMed, CNKI, Wanfang
rTMS neuromodulation	*“repetitive transcranial magnetic stimulation” OR “rTMS” AND “sleep” AND “stroke”*	PubMed, Embase, Cochrane, WoS
Circadian rhythm	*“circadian rhythm” OR “melatonin” OR “light therapy” AND “stroke” OR “brain injury”*	PubMed, Embase, WoS

### 2.3 Inclusion and exclusion criteria

Inclusion criteria: (1) Peer-reviewed original research articles, systematic reviews, and meta-analyses; (2) Studies conducted in human post-stroke populations or experimental models of stroke and sleep; (3) Publications from January 2000 through March 10, 2026; (4) English-language articles and Chinese-language articles with verified translations; (5) Studies reporting at least one sleep disorder outcome (e.g., AHI, PSQI, ISI, prevalence).

Exclusion criteria: (1) Case reports with fewer than 5 participants; (2) Non-peer-reviewed publications (e.g., conference abstracts without full data, editorials, opinion pieces); (3) Studies published before 2000; (4) Duplicate publications of the same dataset (the most comprehensive report retained); (5) Studies in which the stroke diagnosis was not confirmed by neuroimaging.

### 2.4 Evidence quality assessment

Given the narrative design of this review, we did not perform a formal systematic search, GRADE-based evidence grading, or PRISMA flow diagram construction. Instead, to balance synthesis quality with domain breadth, we prioritized randomized controlled trials, meta-analyses, peer-reviewed studies in high-impact journals, and established clinical guidelines, while selectively incorporating observational studies and expert consensus where higher-level evidence was lacking. Given the marked heterogeneity in sleep disorder definitions, assessment tools, and outcome measures across studies, findings were integrated via narrative synthesis rather than quantitative pooling, with the strength of supporting evidence explicitly contextualized throughout the text by denoting specific study designs (e.g., “a randomized trial showed…,” “a meta-analysis demonstrated…”).

## 3 Classification and epidemiology of post-stroke sleep disorders

### 3.1 Overview of sleep disorder classification in stroke

Post-stroke sleep disorders encompass a broad spectrum of conditions, including obstructive sleep apnea, central sleep apnea, insomnia, hypersomnia, parasomnias, sleep-related movement disorders, and circadian rhythm disturbances. The International Classification of Sleep Disorders (ICSD-3) provides the standard diagnostic framework, though application in stroke populations may be complicated by neurological symptoms and medication effects.

Among these conditions, OSA represents the most extensively studied and clinically significant sleep disorder in stroke populations. OSA is characterized by recurrent complete or partial upper airway collapse during sleep, resulting in repetitive apneas and hypopneas, oxygen desaturation, sleep fragmentation, and sympathetic activation. The apnea-hypopnea index (AHI), defined as the average number of apneas and hypopneas per hour of sleep, serves as the primary diagnostic and severity metric. An AHI ≥ 5 events/h indicates at least mild OSA, while AHI ≥ 30 events/h denotes severe disease (26).

Central sleep apnea (CSA) may also occur following stroke, particularly in patients with heart failure or brainstem involvement. CSA results from temporary loss of respiratory drive due to dysfunction of central nervous system respiratory centers, rather than upper airway obstruction. Cheyne-Stokes respiration, a characteristic breathing pattern of alternating crescendo-decrescendo ventilation separated by central apneas, is commonly observed in stroke patients with cardiovascular comorbidities (27).

Insomnia, defined as difficulty initiating or maintaining sleep with associated daytime impairment, represents another prevalent post-stroke sleep complaint. Unlike OSA, which predominantly involves respiratory mechanisms, insomnia in stroke populations often reflects a combination of neurological damage to sleep–wake regulation centers, psychological factors (depression, anxiety, post-traumatic stress), medication effects, and environmental disturbances (28, 29).

### 3.2 Prevalence of obstructive sleep apnea in stroke

Multiple epidemiological studies have consistently demonstrated extremely high prevalence rates of OSA among stroke patients. A systematic evaluation of hospital-based retrospective studies revealed that sleep disorders affect a substantial proportion of stroke patients, with OSA representing the predominant diagnosis (30). The Brain Attack Surveillance in Corpus Christi (BASIC) project, a population-based study conducted in the United States, documented OSA (defined as respiratory event index ≥10) in 62.8% of ischemic stroke patients within a median of 4 days after stroke onset (21). Similarly, international studies have reported OSA prevalence ranging from 40 to 78% in stroke cohorts, substantially exceeding rates observed in age- and sex-matched community samples (31, 32).

According to the 2023 Chinese expert consensus, the prevalence of insomnia symptoms that do not meet the full diagnostic criteria reaches 47.1% in the acute phase of stroke, whereas 32.5% of patients fulfill the diagnostic criteria for insomnia disorder (33). These figures underscore the substantial burden of sleep disturbances in the early post-stroke period within the Chinese population and highlight the pressing need for routine screening and early intervention.

A landmark meta-analysis of prospective cohort studies demonstrated a significant positive association between OSA and both fatal and non-fatal stroke, with a pooled relative risk of 2.15 (95% CI, 1.42–3.24) (34). This association persisted after adjustment for conventional vascular risk factors including hypertension, diabetes, and obesity, consistent with an independent causal relationship, although residual confounding cannot be excluded. Importantly, the relationship appears dose-dependent, with greater OSA severity conferring progressively higher stroke risk.

Population-based studies have further elucidated the epidemiology of OSA in stroke-free populations. The Wisconsin Sleep Cohort Study found that OSA (AHI ≥ 15 events/h) conferred a 3.0-fold increased risk of ischemic stroke in middle-aged men after controlling for body mass index, hypertension, and other confounders (35). Similar findings have been replicated across diverse ethnic groups and geographic regions, establishing OSA as a robust and generalizable stroke risk factor (34, 36).

The temporal dynamics of post-stroke OSA warrant particular attention. While OSA severity may fluctuate during the acute post-stroke period due to factors including neurological impairment, medication effects, and supine sleep positioning, prevalence remains elevated even in chronic stroke stages. Approximately one-third of stroke survivors continue to meet criteria for moderate-to-severe OSA beyond 3 months post-stroke, indicating persistent risk requiring ongoing management (37).

### 3.3 Prevalence of other sleep disorders post-stroke

Beyond OSA, other sleep disorder categories demonstrate substantial prevalence in stroke populations. A systematic review and meta-analysis of 279 prospective cohort studies, encompassing 117,440 stroke participants, reported that the pooled prevalence of sleep disturbance was 59.9% (95% CI, 53.9–63.9%) (38). This figure encompasses multiple sleep disorder diagnoses and subjective sleep complaints, highlighting the breadth of sleep-related morbidity in stroke survivors.

Insomnia symptoms following stroke have been reported in 20–56% of patients across various studies (4, 28). A cross-sectional investigation conducted in Iran found that 84% of stroke patients met criteria for poor sleep quality as assessed by the Pittsburgh Sleep Quality Index (PSQI) (29). Contributing factors include lesion location (particularly involving brainstem and thalamic regions), post-stroke depression, pain, medication effects, and environmental disruptions during hospitalization.

Sleep-related movement disorders, particularly restless legs syndrome (RLS) and periodic limb movement disorder (PLMD), occur with elevated frequency after stroke. RLS, characterized by uncomfortable leg sensations and an urge to move that worsens during rest and evening hours, has been reported in 8–27% of stroke patients compared with 5–10% in the general population (39). Lesions affecting dopaminergic pathways, spinal cord, or peripheral nervous system may contribute to post-stroke RLS.

Hypersomnia, defined as excessive daytime sleepiness disproportionate to inadequate nocturnal sleep, affects 10–25% of stroke survivors (40). The presence of hypersomnia in post-stroke patients often signals underlying sleep-disordered breathing, hypothalamic damage, or depression-related fatigue. Stroke patients with hypersomnia demonstrate worse rehabilitation participation and functional outcomes compared to those without (41).

Sleep disorders demonstrate bidirectional associations with post-stroke depression (PSD), creating complex interrelationships that substantially impact recovery. A cross-sectional study conducted in Vietnam found that PSD was present in 38% of stroke patients and was significantly associated with decreased sleep quality and increased fatigue (22). Depression and sleep disturbance may influence each other through shared neurobiological pathways and behavioral mechanisms, creating synergistic negative effects on rehabilitation engagement and functional recovery.

### 3.4 Risk factors for post-stroke sleep disorders

Multiple factors influence the development and severity of sleep disorders following stroke. Older age represents a consistent risk factor for both OSA and other sleep disturbances, reflecting age-related changes in sleep architecture, upper airway physiology, and neurological control mechanisms (9). Male sex is associated with elevated OSA risk in the general population, though this sex differential may attenuate following stroke due to hormonal changes and differential stroke locations.

Obesity, as assessed by body mass index (BMI) and neck circumference, represents the strongest modifiable risk factor for OSA. Adiposity surrounding the upper airway increases tissue pressure and promotes collapse during sleep, while visceral obesity contributes to systemic inflammation and cardiovascular disease risk (42). Weight gain following stroke may worsen pre-existing OSA or precipitate new-onset disease.

Stroke characteristics significantly influence sleep disorder risk. Lesion location affects sleep architecture and regulation through damage to specific neural structures. Brainstem lesions directly disrupt sleep–wake regulation centers and respiratory control networks. Thalamic and basal forebrain lesions impair arousal mechanisms and circadian regulation. Right hemisphere strokes may preferentially affect REM (rapid eye movement) sleep, while left hemisphere lesions more commonly impact slow-wave sleep (43).

Stroke severity, as quantified by the National Institutes of Health Stroke Scale (NIHSS), correlates positively with sleep disorder severity. More severely affected patients typically exhibit greater sleep architecture disruption, reflecting larger lesion volumes and more extensive neurological impairment (39). Functional dependency in activities of daily living further compounds sleep problems through environmental factors, caregiver burden, and reduced opportunities for physical activity.

Conventional vascular risk factors including hypertension, diabetes, atrial fibrillation, and smoking independently predict post-stroke sleep disorders. These associations reflect shared pathophysiological mechanisms, particularly endothelial dysfunction and systemic inflammation, underlying both vascular disease and sleep-disordered breathing (44). Additionally, some cardiovascular medications, particularly sedating antihypertensives, may directly affect sleep architecture. Stroke type may influence sleep disorder risk and expression, though most studies have focused on ischemic stroke, with limited data available for hemorrhagic stroke.

**Figure 1:**
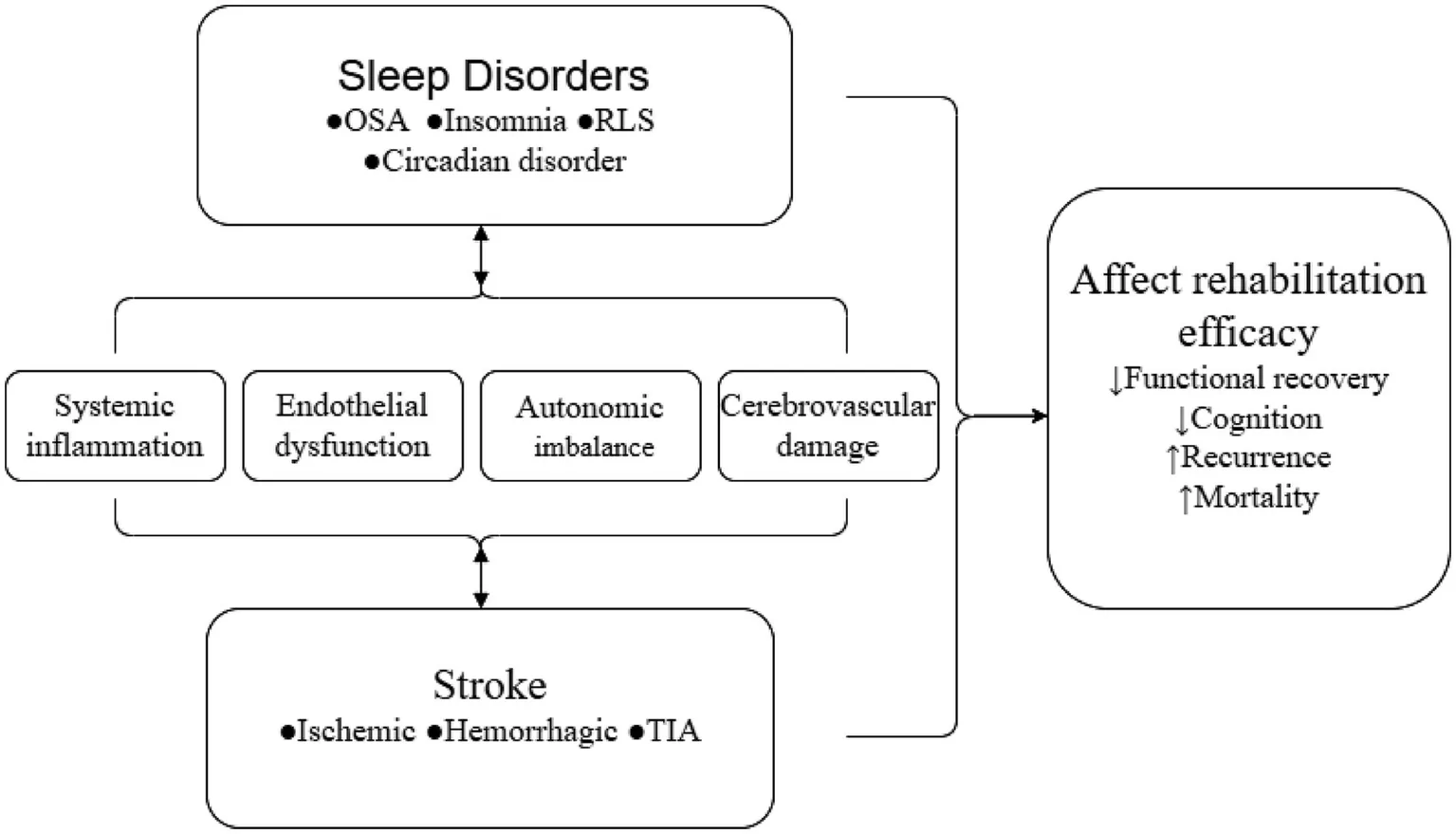
Bidirectional pathophysiological mechanism between sleep disorders and stroke.

The bidirectional pathophysiological mechanism between sleep disorders and stroke is shown in Figure 1.

## 4 Pathophysiological mechanisms linking sleep disorders and stroke

The mechanistic evidence summarized in this section is derived predominantly from studies of ischemic stroke, as hemorrhagic stroke is substantially underrepresented in the sleep-disorder literature. For hemorrhagic stroke, the acute effects of hematoma expansion, mass effect, and elevated intracranial pressure may produce a different profile of sleep disruption, and the pathways linking sleep disorders to secondary brain injury may not be identical to those documented in ischemic stroke. However, dedicated mechanistic studies in hemorrhagic stroke populations remain scarce. Therefore, while the following mechanisms are well supported in the context of ischemic stroke, their applicability to hemorrhagic stroke should be interpreted with caution, and we have noted this limitation where appropriate throughout the discussion.

### 4.1 Intermittent hypoxia and oxidative stress

In OSA, recurrent airway obstruction produces intermittent hypoxia—cyclic fluctuations between normoxia and hypoxia with each apnea event. This pattern, distinct from sustained hypoxia, triggers pro-atherogenic cascades. Intermittent hypoxia stimulates ROS production via NADPH oxidase, mitochondrial dysfunction, and xanthine oxidase pathways (45). The ensuing oxidative stress damages endothelium, promotes LDL oxidation, and fuels inflammation that accelerates atherosclerosis. Animal models show upregulated vascular adhesion molecules, increased blood–brain barrier (BBB) permeability, and enhanced thrombosis (46).

The hypoxia-inducible factor (HIF) pathway mediates adaptive responses. HIF-1α accumulation during hypoxia induces genes for erythropoiesis, angiogenesis, and glycolysis; yet chronic activation may paradoxically foster inflammation and cardiovascular dysfunction (47). In stroke, HIF-1α confers acute neuroprotection but sustained activation may become detrimental. Intermittent-hypoxia-driven NF-κB activation and pro-inflammatory cytokine release (TNF-α, IL-6, CRP) are consistently elevated in OSA, contributing to insulin resistance, endothelial dysfunction, and cardiovascular risk (48). These mediators can cross the damaged BBB after stroke, potentially amplifying secondary injury.

### 4.2 Systemic inflammation and endothelial dysfunction

Chronic systemic inflammation is a hallmark of OSA and a mechanistic link to stroke. Repetitive hypoxia-reoxygenation cycles generate oxidative stress that sustains a self-perpetuating inflammatory loop.

Matrix metalloproteinase-9 (MMP-9) is a key mediator in OSA–stroke interplay. Upregulated by intermittent hypoxia, MMP-9 destabilizes atherosclerotic plaques, disrupts the BBB, and promotes neuronal injury (49). Elevated MMP-9 levels in OSA patients correlate with cardiovascular events.

Endothelial dysfunction—impaired NO bioavailability and heightened vascular reactivity—directly results from OSA-related oxidative stress and inflammation (50). Reduced NO and increased endothelin-1 favour vasoconstriction, hypertension, and a prothrombotic state. Endothelial dysfunction predicts future cardiovascular events and may mediate the OSA–stroke relationship.

Vascular adhesion molecules (ICAM-1, VCAM-1) rise with inflammatory stimuli in OSA, facilitating leukocyte adhesion and trans-endothelial migration—early steps in atherogenesis (51).

### 4.3 Autonomic dysregulation and hemodynamic changes

OSA is associated with profound autonomic changes: sympathetic overactivity, reduced heart rate variability, and apnoea-linked blood pressure surges. These disturbances persist during wakefulness, driving hypertension and cardiovascular risk (52). Acute apnoea triggers intrathoracic pressure swings, reduced venous return, and arrhythmias; airway reopening elicits sympathetic surges that stress the cardiovascular system.

Baroreflex sensitivity is impaired in OSA, possibly related to chronic intermittent hypoxia and sustained sympathetic activation (53). Impaired baroreflex buffering increases blood pressure variability and compromises cerebrovascular autoregulation, potentially raising stroke risk. OSA also shows bidirectional links with atrial fibrillation: intermittent hypoxia and atrial remodelling promote arrhythmogenesis, while atrial fibrillation can precipitate central sleep apnoea; their co-existence synergistically elevates stroke risk (54).

### 4.4 Glymphatic system dysfunction and neurotoxic clearance

The glymphatic system—a brain-wide waste-clearance network reliant on perivascular aquaporin-4 (AQP4) channels—clears neurotoxins including amyloid-β and tau (55). Sleep disruption, including that caused by OSA, has been associated with impaired glymphatic clearance in animal models and may worsen post-stroke injury.

AQP4, highly expressed on astrocytic end-feet, facilitates CSF-interstitial fluid exchange (56). AQP4 deletion abolishes glymphatic clearance and accelerates amyloid-β deposition. After stroke, AQP4 expression and polarization are altered, compounding clearance failure.

Sleep fragmentation reduces slow-wave sleep (SWS)—the period of maximal glymphatic activity. During SWS, the extracellular space expands ~60%, and convective interstitial fluid flow rises, promoting solute clearance (57). Fragmented sleep limits SWS accumulation and thus glymphatic efficiency. In stroke, impaired clearance may delay removal of excitotoxic glutamate and inflammatory cytokines from peri-infarct regions, prolonging secondary injury (58). Conversely, sleep optimization might enhance clearance and aid recovery. Glymphatic dysfunction also links to cerebral small vessel disease (CSVD), creating a vicious cycle: waste accumulation promotes CSVD, while CSVD-damaged perivascular pathways further impair clearance (59).

### 4.5 Neuroinflammation and microglial activation

Stroke triggers robust neuroinflammation—microglial activation, cytokine release, and leukocyte infiltration—that shapes injury and repair. Sleep disorders may modulate these responses, potentially shifting the balance towards harm or benefit.

Microglia adopt phenotypes ranging from pro-inflammatory (M1) to anti-inflammatory (M2) (60). M1 cells release TNF-α, IL-1β, and IL-6, exacerbating injury; M2 cells produce neurotrophic and anti-inflammatory factors that support repair. Sleep deprivation enhances microglial activation in experimental models, potentially priming the brain for exaggerated responses to subsequent stroke (61). This may relate to loss of noradrenergic inhibitory input from the locus coeruleus.

Systemic inflammation in OSA (elevated CRP, IL-6, TNF-α) can potentiate neuroinflammation via circumventricular organ leakage, BBB disruption, and increased adhesion molecule expression (62), creating a bidirectional peripheral–central crosstalk that amplifies brain injury.

### 4.6 Neuroplasticity and neural network remodelling

Sleep is essential for memory consolidation, synaptic plasticity, and network reorganisation—all critical for stroke recovery. Disrupted sleep may impair these processes and may limit rehabilitation gains.

During SWS, hippocampal–neocortical dialogue consolidates memories, with replay of waking neural activity strengthening synaptic connections (63). REM sleep supports emotional and procedural learning. Post-stroke sleep architecture disruption may compromise these plastic mechanisms.

Repetitive transcranial magnetic stimulation (rTMS) enhances stroke recovery partly through neuroplasticity—high-frequency rTMS induces long-term potentiation (LTP)-like effects (64). Combining neuromodulation with sleep optimisation is a promising avenue. Resting-state fMRI reveals altered functional connectivity in stroke patients with sleep disorders, affecting both lesioned and contralesional networks, suggesting widespread dysfunction that may mediate cognitive impairment (65).

## 5 Sleep disorders are associated with poorer post-stroke recovery across multiple domains

As noted in Section 4, the available evidence is drawn predominantly from ischemic stroke studies; hemorrhagic stroke remains substantially underrepresented in the sleep-disorder and recovery literature. The mechanisms and trajectories by which sleep disorders affect recovery may differ between stroke types, but current data do not permit a robust differentiation. Thus, the following findings should be interpreted as applying primarily to ischemic stroke populations, a limitation we acknowledge in Section 8.

### 5.1 Impaired motor recovery and rehabilitation engagement

Accumulating evidence demonstrates that sleep disorders, particularly OSA and insomnia, adversely affect functional recovery following stroke. Patients with sleep-disordered breathing tend to demonstrate worse motor function, slower gait velocity, and greater disability compared to those without (66). The effects appear dose-dependent, with more severe OSA conferring progressively worse functional outcomes.

The Sleep Effects on Post-stroke Rehabilitation (SLEEPR) Study, a prospective cohort investigation following individuals from inpatient rehabilitation through 90 days post-stroke, aims to characterize the impact of non-OSA sleep disorders on recovery (67). Preliminary findings suggest that sleep disturbances at baseline predict worse functional outcomes independent of stroke severity and other confounders.

Balance and gait function, critical determinants of post-stroke independence and fall risk, are particularly vulnerable to sleep disorder effects. A retrospective study of 140 first-ever stroke patients found that those with sleep disturbances demonstrated worse Berg Balance Scale (BBS) scores and slower 10-meter walk velocities compared to patients without sleep disturbances, with sleep disturbance emerging as an independent predictor of balance and gait outcomes (21).

Mechanisms linking sleep disorders to impaired functional recovery include reduced participation in rehabilitation activities, diminished neuroplasticity, increased fatigue, and amplified inflammatory responses (41). Patients with post-stroke hypersomnia may demonstrate reduced motivation and engagement in therapy sessions, limiting opportunities for skill acquisition and functional improvement.

A prospective multicenter study of 69 basal ganglia stroke patients with OSA found that those randomized to continuous positive airway pressure (CPAP) therapy demonstrated significant improvements in Fugl-Meyer Assessment (FMA), Barthel Index (BI), and Mini-Mental State Examination (MMSE) scores over 24 months compared to controls (68). These findings suggest that treatment of sleep-disordered breathing may enhance functional recovery trajectories.

### 5.2 Cognitive decline: attention, executive function, and memory deficits

Sleep disorders exert substantial negative impacts on cognitive function following stroke, affecting attention, executive function, memory, and processing speed. The combined effects of stroke-related brain injury and sleep-related cognitive impairment create particularly vulnerable populations.

A comparative cross-sectional study of 41 chronic stroke patients and 41 age-matched controls found that stroke survivors demonstrated worse performance on measures of sleep quality and multiple cognitive domains, with significant correlations between sleep parameters and cognitive test scores (69). Sleep quality accounted for substantial variance in cognitive performance beyond that explained by stroke severity and location.

The relationship between OSA and cognitive dysfunction in stroke patients has been investigated in multiple studies. OSA-related intermittent hypoxia, sleep fragmentation, and cerebral hypoperfusion likely contribute to cognitive impairment through cumulative neuronal injury in regions critical for memory and executive function (70). Neuroimaging studies have documented reduced hippocampal volumes and white matter integrity in OSA patients.

A large registry-based study found that OSA was associated with a 2.3-fold increased risk of dementia over median 5-year follow-up, with stronger associations observed in younger patients (71). Stroke patients with comorbid OSA may face particularly elevated long-term cognitive decline risk, warranting aggressive screening and treatment.

The Sleep and Cognitive Function in Chronic Stroke study demonstrated that the relationship between sleep and cognition varies by cognitive domain, with associations particularly strong for attention and processing speed (69). These findings suggest domain-specific mechanisms linking sleep disorders to particular cognitive impairments.

### 5.3 Exacerbation of depression, anxiety, and reduced quality of life

Post-stroke depression (PSD) demonstrates bidirectional relationships with sleep disorders, creating mutually reinforcing negative cycles that compound suffering and impede recovery. Depression and sleep disturbance share underlying neurobiological mechanisms and may potentiate each other through neurochemical and behavioral pathways.

The Vietnamese cross-sectional study found that PSD affected 38% of 157 stroke patients and was significantly associated with decreased sleep quality and increased fatigue severity (22). Both sleep quality and fatigue emerged as independent predictors of PSD in multivariate analysis, highlighting the importance of addressing sleep in depression prevention efforts.

Anxiety symptoms also demonstrate significant associations with sleep disorders in stroke populations. Social support, through its relationships with anxiety and depression, exerts indirect effects on sleep quality, suggesting potential intervention targets for improving sleep outcomes (72).

Sleep disorders following stroke are associated with diminished health-related quality of life across multiple domains. Patients with sleep-disordered breathing report worse physical functioning, reduced role limitations due to physical and emotional problems, decreased vitality, and poorer general health perceptions (73). These quality of life decrements compound the burden of stroke-related disability.

Apathy and hypersomnia following stroke represent particularly concerning constellations of symptoms that substantially impair rehabilitation engagement. A retrospective review of 213 acute rehabilitation patients found that apathy affected 21% and hypersomnia affected 5.6% of the cohort, with both conditions associated with discharge to nursing home rather than home and significantly lower Functional Independence Measure (FIM) scores (42).

### 5.4 Elevated risk of stroke recurrence and mortality

Untreated sleep disorders, particularly OSA, are associated with increased risk of recurrent stroke and mortality. This association reflects both the direct vascular effects of sleep-disordered breathing and the indirect consequences of worsened recovery outcomes.

The BASIC project demonstrated that sleep-disordered breathing accounted for significant proportions of ethnic disparities in stroke recurrence and mortality, suggesting that targeted OSA treatment might reduce these inequities (20). Mexican American stroke patients experienced disproportionately higher rates of sleep-disordered breathing and worse outcomes compared to non-Hispanic White patients.

Long-term observational studies have documented increased all-cause and cardiovascular mortality among stroke patients with OSA compared to those without (74). The mechanisms likely include sustained exposure to intermittent hypoxia, heightened inflammatory state, and hypertension persisting through the post-stroke period.

Treatment of OSA with CPAP may reduce stroke recurrence risk. A meta-analysis of cohort studies found that CPAP treatment was associated with lower stroke incidence, with pooled relative risks of 0.27 (95% CI: 0.14–0.53) for stroke and 0.54 (95% CI: 0.38–0.75) for cardiovascular events (75). However, randomized trial evidence remains limited and conflicting.

### 5.5 Interventions targeting sleep disorders may improve stroke recovery outcomes

The recognition that sleep disorders represent modifiable risk factors with substantial impacts on stroke outcomes creates opportunities for targeted interventions to improve recovery. Treatment of sleep disorders may simultaneously address multiple pathophysiological pathways and functional domains.

Pharmacological approaches to post-stroke insomnia include sedative-hypnotics, melatonin receptor agonists, and antidepressants with sleep-promoting properties. However, medication use in stroke populations requires careful consideration of stroke mechanism (hemorrhagic vs. ischemic), fall risk, respiratory depression potential, and cognitive side effects (76).

Non-pharmacological interventions including cognitive behavioral therapy for insomnia (CBT-I) offer promising alternatives without medication-related risks. CBT-I addresses maladaptive sleep-related cognitions and behaviors, targeting underlying mechanisms of insomnia rather than simply masking symptoms (77). Adaptation of CBT-I for stroke populations has shown efficacy in improving sleep outcomes.

Lifestyle modifications including weight management, physical activity promotion, and sleep hygiene optimization provide foundational approaches to sleep disorder management (78). These interventions carry minimal risks and offer benefits extending beyond sleep to cardiovascular health and functional capacity.

Environmental modifications in inpatient rehabilitation settings can substantially improve sleep quality. The SIESTA (Sleep for Inpatients: Empowering Staff to Act) protocol, adapted for stroke rehabilitation settings, addresses noise, lighting, care interruptions, and other environmental barriers to sleep optimization (79). Implementation of sleep-promoting protocols may enhance both sleep quality and rehabilitation outcomes.

### 5.6 Circadian rhythm disruption impairs post-stroke motor and cognitive function

Circadian rhythm disturbances represent an increasingly recognized but understudied category of post-stroke sleep disorders. The suprachiasmatic nucleus (SCN) of the hypothalamus serves as the master circadian pacemaker, precisely coordinating daily biological rhythms in sleep–wake cycles, hormone secretion (such as melatonin and cortisol), body temperature, and autonomic functions (43). When stroke lesions affect the hypothalamus, thalamus, basal forebrain, or brainstem circuits, they can directly impair central or peripheral circadian timekeeping mechanisms (77). Simultaneously, secondary environmental and behavioral factors—including prolonged bed rest, reduced physical activity, lack of ambient natural light exposure, irregular daily routines, and frequent medical interventions—further aggravate circadian misalignment (80).

Although comprehensive epidemiological data remain limited, actigraphy and observational studies suggest that circadian misalignment is highly prevalent during the acute and subacute phases of stroke, where it significantly correlates with post-stroke apathy, fatigue, and delayed cognitive recovery (81). Key drivers of post-stroke circadian dysregulation include:

1. *Light and Environmental Deprivation*: insufficient morning sunlight exposure combined with nighttime nocturnal nursing care disruptions in acute/rehabilitation units;
2. *Attenuated Circadian Zeitgebers*: decreased physical activity, daytime napping, and cognitive impairment leading to temporal disorientation;
3. *Neuroendocrine Disruption*: blunting or delayed peak onset of endogenous nocturnal melatonin secretion during the acute stroke phase (82);
4. *Comorbid Sleep Disorders*: coexisting obstructive sleep apnea (OSA) or insomnia disrupting the physiological consolidation of sleep–wake architecture.

## 6 Screening and diagnostic tools for post-stroke sleep disorders

Optimal management of sleep disorders in stroke requires coordinated multidisciplinary care involving neurologists, sleep specialists, physiatrists, psychiatrists, psychologists, nurses, and therapists. This collaborative approach addresses the complex, multifaceted nature of sleep disorders in this population.

Systematic screening represents the essential first step, as sleep disorders remain frequently unrecognized in stroke populations.

Given the high prevalence and prognostic significance of post-stroke sleep disorders, routine screening is strongly recommended. A stepwise approach—starting with brief general sleep questionnaires, followed by disorder-specific screening instruments, and confirmed by objective diagnostic tools where indicated—is both practical and evidence-based. The Pittsburgh Sleep Quality Index (PSQI) serves as the most widely used generic measure of sleep quality, while the Insomnia Severity Index (ISI) quantifies insomnia symptom burden. For specific sleep disorder subtypes, a range of validated screening questionnaires and objective diagnostic modalities have been employed in stroke populations, as summarized in Table 2. Definitive diagnosis of most sleep disorders relies on overnight polysomnography (PSG) or home sleep apnea testing (HSAT) for sleep-disordered breathing, video-PSG fAor RBD, and multiple sleep latency testing (MSLT) for objective confirmation of excessive daytime sleepiness. Actigraphy is particularly useful for circadian rhythm disorders and for patients with poor sleep perception.

**Table 2:** Screening and diagnostic tools for major post-stroke sleep disorders.

Sleep disorder subtype	Screening tools (questionnaires/brief assessments)	Diagnostic tools (objective/confirmatory)
Sleep-Disordered Breathing (SDB)	STOP-BANG/STOP-BANG2; Berlin Questionnaire (BQ); 4-Variable Questionnaire (4-VQ); Sleep List; Epworth Sleepiness Scale (ESS); overnight pulse oximetry (83, 119–123)	Polysomnography (PSG)—gold standard; Home Sleep Apnea Testing (HSAT); Adaptive servo-ventilation (ASV) for CSA (88, 124, 125)
Insomnia	Insomnia Severity Index (ISI); Pittsburgh Sleep Quality Index (PSQI); sleep diary (108, 126, 127)	PSG (for refractory or coexisting SDB/PLMS); Actigraphy (for circadiAan patterns) (128–130)
Excessive Daytime Sleepiness (EDS)	ESS; Stanford Sleepiness Scale (SSS); Fatigue Severity Scale (FSS) (125, 131, 132)	Multiple Sleep Latency Test (MSLT); Maintenance of Wakefulness Test (MWT); PSG (to exclude other sleep disorders) (133–135)
RLS/PLMS	International RLS Rating Scale (IRLS); RLS Quality of Life Questionnaire (RLS-QoL); clinical criteria (ICSD-3) (136, 137)	PSG (PLM index ≥ 15/h); Suggested Immobilization Test (SIT) (138–140)
RBD	RBD Screening Questionnaire (RBDSQ); RBD Questionnaire-Hong Kong (RBDQ-HK); RBD Single-Question Screen (RBD1Q); Mayo Sleep Questionnaire (MSQ) (141–144)	Video-Polysomnography (video-PSG)—gold standard (145, 146)
CRSWDs	Sleep diary; PSQI; ESS (to differentiate daytime vs. nighttime symptoms) (108, 122, 125)	Actigraphy (≥ 7 days); Dim Light Melatonin Onset (DLMO); PSG (to exclude coexisting disorders) (147–149)

The STOP-Bang questionnaire and Berlin Questionnaire provide validated screening tools for OSA, while the PSQI and Insomnia Severity Index assess insomnia symptoms (83). The Ottawa Model for Research Use provides a framework for addressing gaps between evidence and practice in post-stroke sleep management (8). This model emphasizes identification of barriers to knowledge translation, adaptation of interventions to local contexts, assessment of implementation outcomes, and sustainability planning. Simple screening approaches are particularly important given time constraints in busy clinical settings.

## 7 Management strategies for sleep disorders in stroke

### 7.1 Continuous positive airway pressure therapy for OSA

Continuous positive airway pressure (CPAP) therapy represents the established first-line treatment for moderate-to-severe OSA in the general population and is increasingly recognized as essential for stroke patients with sleep-disordered breathing. CPAP maintains upper airway patency through positive pressure delivered via nasal or oronasal mask, preventing collapse during sleep.

Multiple studies have demonstrated improvements in sleep parameters, blood pressure, and cardiovascular outcomes with CPAP adherence in stroke populations (84, 85). Existing study and investigations indicate that CPAP treatment reduces AHI, improves oxygen saturation, and decreases sympathetic activation in post-stroke patients.

Beyond sleep-specific benefits, CPAP therapy may enhance neurological recovery, although some randomized trials have reported null effects on functional and cognitive outcomes. A prospective multicenter trial randomized 69 basal ganglia stroke patients with OSA to receive CPAP or no CPAP in addition to standard rehabilitation (68). At 6 months, the CPAP group demonstrated significantly greater improvements in FMA (motor function), MMSE (cognitive function), BI (activities of daily living), and Hamilton Anxiety and Depression Scale scores compared to controls.

Despite demonstrated benefits, CPAP adherence remains a major challenge in post-stroke populations. Stroke-related cognitive impairment, facial weakness, dysphagia, and motor deficits may impede mask fitting and device operation, thereby limiting CPAP tolerance in affected patients. A meta-analysis of randomized trials reported that CPAP use after stroke is generally acceptable once the treatment is tolerated, with a mean nightly usage of approximately 4.53 h across studies; however, dropout rates were higher with CPAP (odds ratio 1.83), suggesting lower adherence particularly among individuals with more severe stroke symptoms (or higher stroke severity) (86).

Innovative approaches to improve CPAP adherence in stroke populations include intensive education programs, early initiation during acute hospitalization, telemonitoring with automated feedback, and heated humidification to reduce nasal side effects (87). Specialized interfaces accommodating facial asymmetry or oral breathing may also improve comfort and tolerability.

Alternative positive airway pressure modalities including bilevel-PAP (BPAP) and adaptive servo-ventilation (ASV) may be considered for patients intolerant of standard CPAP. BPAP provides differential pressures for inspiration and expiration, reducing work of breathing. ASV adjusts pressure delivery in response to breathing pattern variations, potentially beneficial for central apneas or complex sleep apnea (88).

Although some randomized controlled trials have reported null effects of CPAP on certain functional and cognitive outcomes in stroke patients, the preponderance of evidence—particularly from studies with good adherence—suggests potential benefits. Given the considerable heterogeneity across existing studies, further large-scale, well-designed randomized trials are needed to definitively establish the impact of CPAP on functional and cognitive recovery after stroke.

### 7.2 Cognitive behavioral therapy for insomnia

Cognitive behavioral therapy for insomnia (CBT-I) represents the gold-standard non-pharmacological treatment for chronic insomnia in general adult populations and has demonstrated efficacy in post-stroke and acquired brain injury populations. CBT-I addresses maladaptive sleep-related cognitions and behaviors through structured therapeutic components.

The core components of CBT-I include sleep restriction therapy, stimulus control, cognitive restructuring, sleep hygiene education, and relaxation training. Sleep restriction therapy limits time spent in bed to actual sleep duration, creating mild sleep deprivation that consolidates sleep and strengthens homeostatic drive. Stimulus control addresses conditioning factors by strengthening associations between bed and sleep while reducing wakeful activities in the sleep environment (89).

A randomized controlled trial of 54 adults with acquired brain injury (ABI) and self-reported sleep disturbances, including stroke patients, compared CBT-I plus treatment as usual (TAU) versus TAU alone (90). The CBT-I plus TAU group demonstrated significantly greater improvements in sleep quality (PSQI), with a large between-group effect size (*d* = 0.924). Secondary outcomes including dysfunctional beliefs about sleep and fatigue also improved.

Adaptations of CBT-I for stroke populations must address cognitive, communication, and physical limitations that may affect treatment delivery. Modifications may include simplified psychoeducation materials, extended treatment duration, caregiver involvement, and telehealth delivery formats (91). Studies support the feasibility and efficacy of adapted CBT-I protocols in stroke rehabilitation settings.

The mechanisms through which CBT-I improves outcomes in stroke likely extend beyond sleep optimization. Reduced insomnia symptoms may decrease depression and anxiety, enhance daytime alertness and rehabilitation participation, improve neuroplasticity through sleep architecture normalization, and reduce systemic inflammation through stress pathway modulation (92).

### 7.3 Pharmacological interventions

Pharmacological management of post-stroke sleep disorders requires careful consideration of stroke type, comorbidities, medication interactions, and side effect profiles. Medications may serve as adjuncts to non-pharmacological approaches or as primary treatments when behavioral interventions are insufficient or unavailable.

Sedative-hypnotics, including benzodiazepines and non-benzodiazepine receptor agonists, are frequently prescribed for insomnia but carry significant risks in stroke populations. These medications increase fall risk through residual sedation, may impair cognitive recovery, and carry dependence potential with withdrawal syndromes upon discontinuation (76, 93). If used, lowest effective doses for shortest durations are recommended.

Melatonin receptor agonists, including agomelatine and ramelteon, offer alternatives with favorable safety profiles. Agomelatine, which additionally possesses antidepressant properties through melatonergic and serotonergic mechanisms, has demonstrated efficacy for post-stroke insomnia with improvements in both sleep quality and mood parameters (94). The sleep-promoting effects of melatonin agonists involve circadian rhythm alignment rather than direct sedation.

The combination of acupuncture with melatonin agonists has shown promise in stroke populations. A randomized trial comparing electroacupuncture, agomelatine, and combination therapy found that all interventions improved sleep efficiency and cognitive function, with combination therapy demonstrating superior efficacy over monotherapies (94). These findings suggest potential synergistic effects of multimodal approaches.

Antidepressants with sedating properties, including trazodone, mirtazapine, and amitriptyline, may address comorbid insomnia and depression in stroke patients. However, these medications carry risks including orthostatic hypotension, cardiac conduction abnormalities, and morning sedation that require monitoring (95). Mirtazapine’s appetite-stimulating effects may benefit stroke patients with malnutrition or dysphagia.

Orexin receptor antagonists, including suvorexant and lemborexant, represent newer medication classes that target the arousal system. By blocking wake-promoting orexin signaling, these medications facilitate sleep onset and maintenance without the dependence risks of traditional sedatives (96). Emerging evidence in general populations suggests efficacy and tolerability, though studies specifically in stroke patients remain limited.

### 7.4 Acupuncture and traditional Chinese medicine

In China, several traditional Chinese medicine (TCM) approaches—including acupuncture and herbal formulations such as modified Suanzaoren decoction and Bailemian capsules—have been investigated for post-stroke sleep disorders, with some studies reporting beneficial effects (34, 97). A meta-analysis of 26 randomized controlled trials, cited in the 2023 Chinese expert consensus, found that acupuncture improved sleep quality in stroke patients with insomnia (34). Certain herbal preparations have also shown preliminary efficacy in acute stroke patients with comorbid insomnia (97). However, systematic reviews have consistently rated the available evidence as low or very low certainty, due to high risk of bias, inadequate blinding, and potential publication bias. Moreover, most studies originate from a single country, limiting the generalizability of their findings to broader international stroke populations. Therefore, while these approaches may be considered in appropriate clinical contexts—particularly for patients who prefer or have access to such therapies—they should not be presented with a level of confidence comparable to established first-line interventions. Future rigorously designed, multicenter, sham-controlled trials are needed to confirm their efficacy and to clarify their mechanisms of action (65).

The acupuncture and TCM evidence cited above is supported by the 2023 Chinese national expert consensus on stroke-related sleep disorders (34), which should be distinguished from international clinical practice guidelines. This consensus provides a Class IIa recommendation (Level B-R evidence) for acupuncture in treating post-stroke insomnia, based on a meta-analysis of 26 randomized controlled trials demonstrating improved sleep quality with a favorable safety profile (34). However, it is important to note that acupuncture and TCM herbal formulations are not addressed in major international guidelines. The 2023 AASM clinical practice guidelines for chronic insomnia in adults do not include acupuncture or TCM due to insufficient evidence meeting their inclusion criteria (98). The 2020 EAN/ERS/ESO/ESRS joint statement on sleep disorders and stroke provides strong recommendations for OSA screening and CPAP therapy but does not mention acupuncture or TCM (5). Similarly, the 2024 AHA Scientific Statement on Sleep Disorders and Brain Health acknowledges the link between sleep disorders and adverse brain health outcomes but does not address these complementary therapies (99). Thus, while the Chinese national consensus offers region-specific guidance, the evidence base for acupuncture and TCM remains confined primarily to studies conducted in Chinese populations, with acknowledged methodological limitations (high risk of bias, inadequate blinding, publication bias) and low-to-very-low certainty ratings in systematic reviews. Readers should interpret these findings with appropriate caution regarding their generalizability to broader international stroke populations.

### 7.5 Physical activity and exercise interventions

Physical activity and structured exercise interventions demonstrate beneficial effects on both sleep quality and stroke recovery, representing promising non-pharmacological approaches to address sleep disorders in this population.

A rat model of ischemic stroke demonstrated that exercise combined with melatonin therapy significantly improved sleep duration, motor function, and cognitive performance compared to either intervention alone or controls (100). Synergistic mechanisms may involve enhanced hippocampal synaptic plasticity, reduced neuroinflammation, and normalized circadian rhythms.

In human studies, aerobic exercise has been associated with improved sleep quality, reduced insomnia symptoms, and enhanced daytime alertness in stroke populations (101). Exercise likely improves sleep through multiple mechanisms including body temperature elevation, stress reduction, circadian entrainment, and modulation of inflammatory and neurochemical pathways.

Timing of exercise relative to sleep may influence effects. Vigorous exercise close to bedtime may interfere with sleep onset due to sympathomimetic effects, while moderate exercise performed earlier in the day generally promotes sleep quality. Morning exercise may be particularly beneficial for circadian alignment in stroke patients with irregular sleep–wake patterns.

### 7.6 Efficacy of neuromodulation techniques for post-stroke sleep disorders

Accumulating evidence supports the use of repetitive transcranial magnetic stimulation (rTMS) for post-stroke sleep disorders (PSSD). A systematic review of 17 randomized controlled trials (1,411 patients) demonstrated that rTMS has been associated with significant improvements in sleep quality in some trials (PSQI mean difference −2.51), anxiety, and even stroke severity (NIHSS MD –2.71) (102). A network meta-analysis of 28 trials (2,353 patients) comparing various electromagnetic stimulation therapies for post-stroke insomnia ranked low-frequency rTMS among the most effective modalities for reducing PSQI scores (103). Another recent network meta-analysis (15 trials, 1,113 patients) specified that low-frequency rTMS over the right dorsolateral prefrontal cortex (DLPFC) yields the best outcomes for both sleep and comorbid depression, whereas high-frequency left-DLPFC stimulation did not outperform pharmacotherapy alone (104). Parallel studies in primary chronic insomnia confirm that rTMS can alter sleep architecture—increasing slow-wave and REM sleep—with long-term benefits exceeding those of medication or psychotherapy (105).

The mechanisms underlying rTMS efficacy in PSSD are only beginning to be understood. Stroke frequently disrupts cortico-subcortical circuits governing arousal and sleep–wake regulation, including the ascending reticular activating system, thalamocortical loops, and the default mode network. Low-frequency rTMS (≤1 Hz) is hypothesised to suppress local cortical hyperexcitability in perilesional or contralesional regions, thereby restoring interhemispheric balance and normalising the hyperarousal state—a core feature of insomnia driven by sensitised locus coeruleus and salience network activity (106). At the neurotransmitter level, rTMS may enhance cortical GABAergic inhibition and reduce glutamatergic tone, facilitating sleep spindle generation and slow-wave oscillations. A particularly intriguing hypothesis involves the bidirectional link between sleep and plasticity: one study found that stroke patients who fell asleep during low-frequency rTMS sessions showed significantly greater upper limb motor gains than those who remained awake, and sleep depth correlated strongly with functional improvement (107). This raises the possibility that rTMS-induced sleep enhancement may prime the brain for rehabilitation by promoting glymphatic clearance, consolidating motor memory, or upregulating neurotrophic factors. Additionally, because post-stroke depression strongly predicts PSSD, rTMS may act indirectly by modulating fronto-limbic mood circuits (108).

Despite promising findings, several limitations warrant attention. First, most PSSD trials have small sample sizes and heterogeneous protocols (frequency, site, duration), hindering protocol standardisation. Second, a meta-analysis of primary insomnia trials cautioned that nearly three-quarters of the observed effect may be attributable to sham-induced placebo responses (109), emphasising the need for rigorous double-blind, sham-controlled designs in stroke populations. Third, mechanistic studies remain sparse—we lack direct evidence linking rTMS-induced neurochemical changes (e.g., GABA/glutamate measured by magnetic resonance spectroscopy) to sleep architecture improvements in stroke patients. Future research should (i) establish optimal rTMS parameters for PSSD using large, multicentre trials, (ii) integrate polysomnography and neuroimaging to dissect direct sleep-regulating effects from indirect mood- or plasticity-mediated pathways, and (iii) investigate whether enhancing sleep during neuromodulation can potentiate motor recovery, thereby offering a dual therapeutic target for post-stroke rehabilitation.

### 7.7 Treatment of movement disorders associated with stroke-related sleep disturbances

Stroke-related sleep disturbances frequently manifest as movement disorders, among which restless legs syndrome (RLS) and periodic limb movement disorder (PLMD) are the most common. These conditions can significantly impair sleep quality, delay functional recovery, and diminish quality of life in post-stroke patients. Effective management requires a combination of pharmacological and non-pharmacological strategies tailored to the individual patient’s condition and stroke context.

Iron deficiency is a well-established and potentially modifiable risk factor for RLS. Current guidelines recommend checking serum ferritin and transferrin saturation in all patients with RLS symptoms (110, 111). For adults with serum ferritin ≤75 ng/mL or transferrin saturation <20%, oral or intravenous iron supplementation is recommended as first-line treatment (110, 111). In post-stroke patients, special attention should be paid to iron stores, as cerebrovascular lesions may further disrupt brain iron metabolism. Ferrous sulfate (325 mg) combined with vitamin C (100–200 mg) at bedtime is a commonly used regimen to enhance iron absorption (112, 113). Intravenous iron is reserved for patients with intolerance to oral preparations or those with more severe deficiencyt (110, 111).

The 2025 American Academy of Sleep Medicine (AASM) clinical practice guideline marks a significant shift in RLS treatment, moving away from dopamine agonists toward gabapentin enacarbil and other gabapentinoid medications as preferred pharmacological agentst (110, 111). Gabapentin enacarbil is recommended when iron supplementation is insufficient or when patients are not iron-deficient (112, 113). These agents modulate calcium channels and reduce neuronal hyperexcitability, providing relief from both sensory and motor symptoms of RLS. Pregabalin represents an alternative option with similar efficacy (4). In post-stroke patients, gabapentinoids may offer additional benefits for comorbid neuropathic pain or anxiety, making them particularly suitable in the stroke rehabilitation setting.

Dopamine agonists (e.g., pramipexole, ropinirole, rotigotine) have historically been first-line therapy for RLS (114, 115). However, the updated guidelines advise caution due to the risk of augmentation—a worsening of symptoms with prolonged use—which is particularly concerning in long-term management (1, 2). Dopaminergic agents may still be considered in acute post-stroke RLS when symptoms are severe and other options have failed, but they should be used at the lowest effective dose for the shortest duration (114, 115). Levodopa, while sometimes effective for PLMD, is generally not recommended for chronic management due to similar augmentation concerns (116).

Non-pharmacological interventions play a complementary role, especially in post-stroke populations where medication side effects may be poorly tolerated. Lifestyle modifications including regular exercise, avoidance of caffeine, alcohol, and nicotine, and sleep hygiene optimization are recommended as foundational strategies (4, 6). Physical therapy and targeted leg stretching before bedtime may reduce symptom severity. For patients with mild-to-moderate symptoms, massage, warm baths, and compression therapy can provide symptomatic relief (6). Addressing comorbid obstructive sleep apnea (OSA) is essential, as untreated OSA can exacerbate both RLS and PLMD symptoms (110, 111).

PLMD frequently co-occurs with RLS, and treatment largely overlaps. As PLMD is diagnosed when limb movements exceed 15 per hour in the absence of RLS symptoms, identification of underlying causes is critical (4). Polysomnography may be required for definitive diagnosis. In post-stroke patients, secondary PLMD should prompt evaluation for concurrent sleep disorders and appropriate treatment of any identifiable cause (114, 116).

In summary, the therapeutic approach to post-stroke RLS and PLMD should be individualized, beginning with iron assessment and supplementation, followed by gabapentinoid therapy as the preferred pharmacological option. Dopaminergic agents remain available but require careful monitoring. Non-pharmacological strategies and management of comorbid sleep disorders are integral components of comprehensive care.

### 7.8 Management of circadian rhythm abnormalities

The clinical management of post-stroke circadian rhythm disturbances relies on a combined non-pharmacological and pharmacological approach:

1. *Morning bright light therapy (BLT)*: exposure to 2,500–10,000 lux for 30 min upon waking anchors the circadian phase and enhances morning arousal. Randomized controlled clinical trials demonstrate that morning BLT effectively reduces sleep onset latency and improves sleep efficiency in patients with post-stroke insomnia (80). Furthermore, dynamic naturalistic lighting systems installed in rehabilitation wards have been shown to significantly alleviate post-stroke fatigue (117).
2. *Exogenous melatonin supplementation*: administration of 0.5–5 mg of melatonin 1–2 h prior to the desired bedtime induces a circadian phase advance and shortens sleep latency. Beyond circadian modulation, melatonin exerts neuroprotective benefits against ischemic brain injury through free radical scavenging and anti-inflammatory properties (117, 118).
3. *Sleep–wake schedule regularization & environmental hygiene*: maintaining consistent bed and wake times, restricting daytime naps to less than 30 min, increasing daytime sunlight exposure, minimizing nighttime light from screens/medical devices, and structuring regular meal and rehabilitation schedules (43).

Current evidence supporting these interventions in stroke populations remains low-to-moderate in quality (GRADE: low-to-moderate quality; Recommendation: may be considered, particularly in subacute rehabilitation settings). Future randomized trials employing polysomnography (PSG) and actigraphy endpoints are necessary to establish optimized, standardized therapeutic regimens.

## 8 Future directions and research priorities

### 8.1 Research gaps and future priorities

Despite growing recognition of the bidirectional relationship between sleep disorders and stroke, several knowledge gaps impede evidence-based practice. First, heterogeneous diagnostic criteria, assessment tools, and outcome measures across studies complicate data synthesis and cross-study comparison. The development of a core outcome set specifically for post-stroke sleep disorder research is urgently needed to standardize future investigations.

Second, the mechanistic interplay between sleep disturbance and stroke recovery remains incompletely understood. While multiple pathways—including intermittent hypoxia, inflammation, and glymphatic dysfunction—have been implicated, their relative contributions in clinical populations and potential interactions are unclear. Translational research linking preclinical models to human recovery trajectories is required. Moreover, the available evidence derives predominantly from ischemic stroke populations, whereas hemorrhagic stroke remains substantially underrepresented in the sleep-disorder and recovery literature. Whether the pathways and magnitude by which sleep disorders affect recovery differ between stroke types is unknown, as dedicated mechanistic and outcome studies in hemorrhagic stroke are scarce. Future research should explicitly differentiate between ischemic and hemorrhagic stroke to determine whether subtype-specific management strategies are warranted.

Third, long-term prospective studies are lacking on the natural history of post-stroke sleep disorders and their sustained impact on functional recovery, cognition, and quality of life beyond the initial rehabilitation phase.

From a clinical trial perspective, high-priority research areas include: determining the optimal timing of CPAP initiation (acute, subacute, or chronic); developing adherence-enhancing strategies; conducting head-to-head comparisons of different OSA treatments; adapting and validating CBT-I for stroke patients; evaluating combined pharmacological and non-pharmacological approaches; and investigating Traditional Chinese Medicine and integrative therapies. Importantly, trials must adopt patient-centred outcomes—such as functional recovery, quality of life, and community reintegration—rather than relying solely on sleep metrics. Extended follow-up is essential given the chronic nature of both post-stroke disability and sleep disorders.

### 8.2 New ways to do better research

New sleep monitoring technologies enable the collection of more detailed, objective, and continuous data in stroke patients. Home-based sleep apnea testing is becoming increasingly sophisticated, and they may eventually match lab-based polysomnography in diagnostic accuracy.

Wearable devices can track sleep, activity, and other physiological signs over long periods, giving us real-world information about sleep–wake patterns. These tools may improve patient adherence to monitoring and capture day-to-day variability that a single night in the lab misses.

Machine learning and artificial intelligence are promising for improving diagnosis, predicting who will respond to treatment, and personalising interventions. Biomarker panels that combine genetic, protein, and clinical data might 1 day help guide individual treatment choices.

### 8.3 Making it work in real-world care

Even when robust evidence supports the efficacy of sleep disorder treatments, their translation into routine clinical practice remains challenging. Implementation science offers a valuable framework for identifying and overcoming the barriers that hinder the integration of sleep management into standard stroke care.

**Figure 2:**
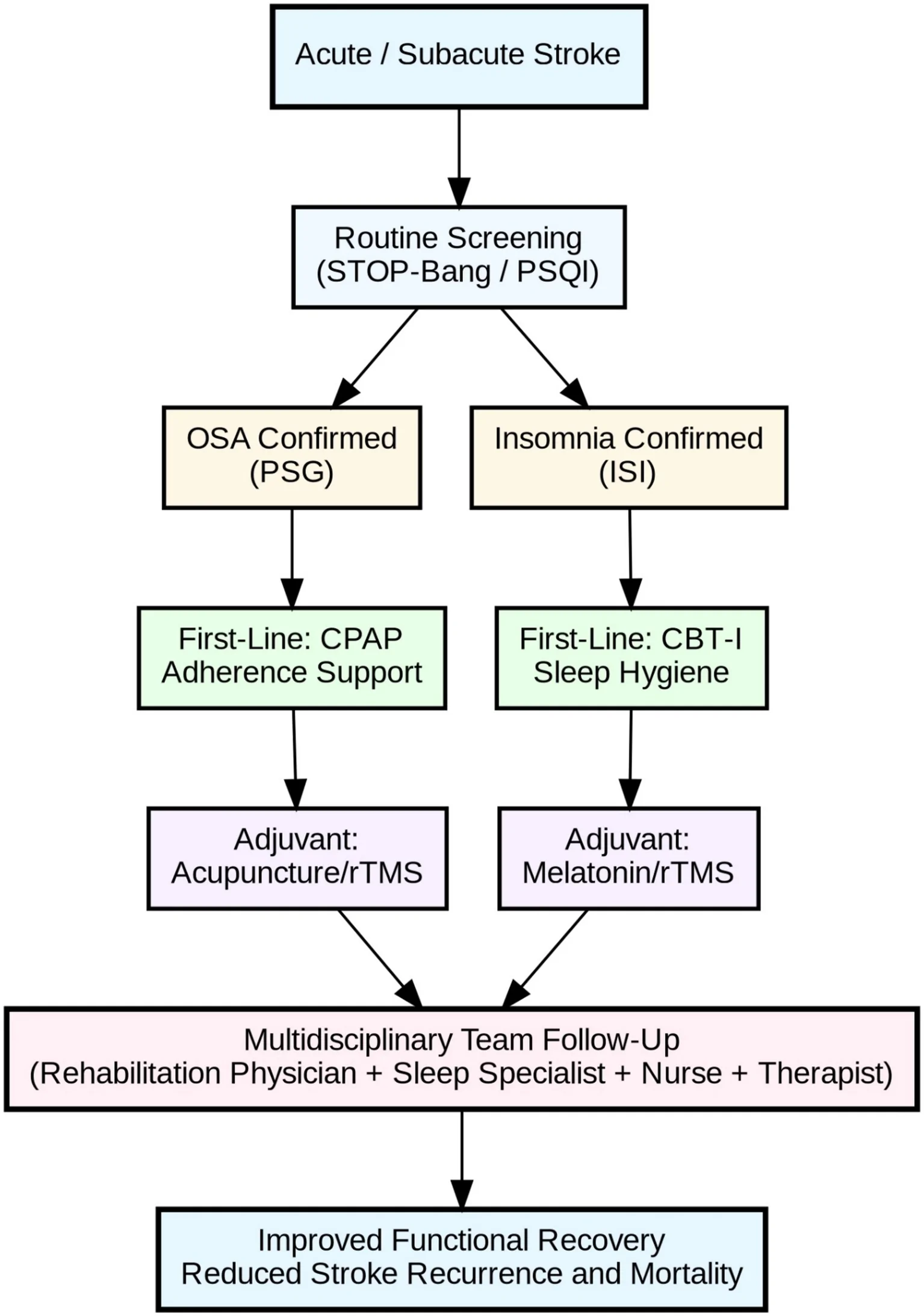
Multidisciplinary management flowchart for sleep disorders after stroke.

Common obstacles to managing sleep after stroke include limited awareness of the issue among stroke care teams, tight clinical workflows that leave insufficient time for sleep assessment, and the absence of standardised assessment protocols. Additional difficulties arise from the challenges of diagnosing sleep disorders in patients with cognitive impairment, restricted access to equipment and resources, and persistent uncertainty regarding which treatment options are most effective. Overcoming these barriers will likely require system-wide changes, such as integrating sleep care into existing clinical pathways, providing decision support tools, automating screening procedures, and using outcome dashboards to track performance. Furthermore, quality improvement methodologies can be employed to continuously refine and optimise sleep care over time. Multidisciplinary management of sleep disorders after stroke is illustrated in Figure 2.

## 9 Conclusion

Sleep disorders represent highly prevalent, clinically significant complications of stroke that profoundly impact recovery outcomes, quality of life, and long-term prognosis. The bidirectional relationship between sleep disturbances and cerebrovascular disease creates mutually reinforcing negative cycles that compound patient suffering and healthcare burden.

Obstructive sleep apnea emerges as the best-characterized sleep disorder-stroke link, with robust epidemiological evidence establishing OSA as an independent stroke risk factor and predictor of adverse post-stroke outcomes. Pathophysiological mechanisms including intermittent hypoxia, systemic inflammation, endothelial dysfunction, and autonomic dysregulation mediate these relationships.

Beyond OSA, insomnia, hypersomnia, sleep-related movement disorders, and other sleep disturbances also affect substantial proportions of stroke survivors. These conditions further impair functional recovery, cognitive function, mood, and quality of life through overlapping and distinct mechanisms.

Evidence-based management strategies exist for post-stroke sleep disorders. CPAP therapy represents the established first-line treatment for OSA, with demonstrated benefits for sleep parameters and potential benefits for neurological recovery, though trial evidence on functional outcomes remains mixed, Cognitive behavioral therapy effectively addresses insomnia symptoms with durable effects and minimal risks. Acupuncture and Traditional Chinese Medicine approaches offer promising complementary options with growing evidence bases.

Despite progress, significant gaps remain in our understanding and management of sleep disorders in stroke populations. Improved screening, mechanistic investigation, rigorous clinical trials, and effective implementation represent priorities for advancing care. The recognition that sleep represents a modifiable target with profound implications for stroke recovery creates opportunities for transformative improvements in patient outcomes.

Future directions should prioritize personalized approaches that integrate sleep optimization into comprehensive stroke rehabilitation programs. Such integration recognizes the fundamental importance of sleep for neurological recovery, neuroplasticity, and human flourishing, while addressing a critically underserved aspect of post-stroke care.

## Glossary

ABI: Acquired Brain Injury
AHI: Apnea-Hypopnea Index
ASV: Adaptive Servo-Ventilation
AQP4: Aquaporin-4
BASIC: Brain Attack Surveillance in Corpus Christi
BBS: Berg Balance Scale
BI: Barthel Index
BMI: Body Mass Index
BPAP: Bilevel Positive Airway Pressure
CBT-I: Cognitive Behavioral Therapy for Insomnia
CPAP: Continuous Positive Airway Pressure
CRP: C-Reactive Protein
CSA: Central Sleep Apnea
CSVD: Cerebral Small Vessel Disease
DLPFC: Dorsolateral Prefrontal Cortex
EA: Electroacupuncture
FIM: Functional Independence Measure
FMA: Fugl-Meyer Assessment
GABA: γ-Aminobutyric Acid
HIF: Hypoxia-Inducible Factor
ICAM-1: Intracellular Adhesion Molecule-1
ICSD-3: International Classification of Sleep Disorders-Third Edition
IL-6: Interleukin-6
ISI: Insomnia Severity Index
LDL: Low-Density Lipoprotein
LTP: Long-Term Potentiation
MMP-9: Matrix Metalloproteinase-9
MMSE: Mini-Mental State Examination
NADPH: Nicotinamide Adenine Dinucleotide Phosphate
NF-κB: Nuclear Factor-κB
NIHSS: National Institutes of Health Stroke Scale
NO: Nitric Oxide
OSA: Obstructive Sleep Apnea
PLMD: Periodic Limb Movement Disorder
PSD: Post-Stroke Depression
PSSD: Post-Stroke Sleep Disorders
PSQI: Pittsburgh Sleep Quality Index
RCTs: Randomized Controlled Trials
REM: Rapid Eye Movement
RLS: Restless Legs Syndrome
ROS: Reactive Oxygen Species
rTMS: Repetitive Transcranial Magnetic Stimulation
SLEEPR: Sleep Effects on Post-stroke Rehabilitation
SIESTA: Sleep for Inpatients: Empowering Staff to Act
SWS: Slow-Wave Sleep
TAU: Treatment As Usual
TCM: Traditional Chinese Medicine
TNF-α: Tumor Necrosis Factor-α
VCAM-1: Vascular Cell Adhesion Molecule-1
